# Incidence and recovery of smell and taste dysfunction in COVID-19 positive patients

**DOI:** 10.1186/s43163-020-00050-0

**Published:** 2020-10-31

**Authors:** Haider Majid Haider Al-Zaidi, Hani Musa Badr

**Affiliations:** 1 College of Medicine, Ibn Sina University of Medical and Pharmaceutical Sciences, Baghdad, Iraq; 2 Gazi Alhariri Hospital, Baghdad, Iraq; 3 Baghdad, Iraq

**Keywords:** Chemosensitive dysfunctions, Anosmia, Taste loss, COVID-19

## Abstract

**Background:**

This study aims to find the chemosensitive dysfunction incidence in COVID-19-positive patients and its recovery.

We collected the data from sixty-five patients, all COVID-19 positive, quarantined in-hospital between 5 April 2020 and 17 May 2020, by a questionnaire distributed in the quarantine ward.

**Results:**

Smell dysfunction appeared in 89.23% with or without other symptoms of COVID-19. 39.66% of them recovered the sense of smell. Taste dysfunction found in 83.08% patients with other COVID-19 symptoms. Only 29.63% of them recovered. The recovery took 1–3 weeks, and most cases recovered within 1 week or less. 18.46% and 15.38% had smell and taste dysfunction, respectively, as the only symptom before COVID-19 confirmation. Most of the chemosensitive dysfunction affected the 4th decade of age in this study.

**Conclusion:**

Chemosensitive dysfunction is associated with coronavirus disease and may be the only symptom that presents the disease. This makes the ENT doctors the first line of contact with the coronavirus. Further objective studies are required to cover chemosensitive dysfunctions, as the recognition of this dysfunction may help the diagnosis of COVID-19, and prevent the spread of this disease.

## Background

There are three types of presentations in COVID-19: asymptomatic, mild upper respiratory tract infection (URTI), and severe systemic disease such as bilateral interstitial pneumonia [1].

Olfactory dysfunction (OD) is already recognized in ENT practice after many viral infections, which can cause OD by inflammation in the sinonasal mucosa and runny nose, with rhinovirus, parainfluenza Epstein-Barr virus, and some coronavirus being the most common viruses [2].

In 2018, Dubé et al. found that the previous form of human coronavirus (HCoV) OC43 reaches the central nervous system through the olfactory epithelium and starts neuropropagation at the olfactory bulbs [3]. Many other recent studies found that smell and taste dysfunctions are seen frequently in COVID-19 patients [4]. Therefore, it is logical to consider the relationship between OD and the new coronavirus disease 2019.

In Iraq, the COVID-19 affected the public life, as in all other affected countries, and since the pandemic, there was an increase in the reported cases of OD and taste loss in hospitals and private clinics.

Thus, we are investigating the incidence in OD and taste disorders as an isolated symptom of coronavirus disease in Iraqi patients.

## Methods

We collected the data from sixty-five patients, all COVID-19 positive, quarantined in quarantine hospital between 5 April 2020 and 17 May 2020, by a questionnaire distributed the quarantine ward.

The patients were included if they were proved to be positive for COVID-19, and they are fully conscious and had willingly given the formal consent of being enrolled in the research.

We excluded patients who had olfactory problems before January 2020.

## Results

Sixty-five COVID-19-positive patients, 27 males (41.54 %) and 38 females (58.46 %), average age 41.2 years

**Fig. 1:**
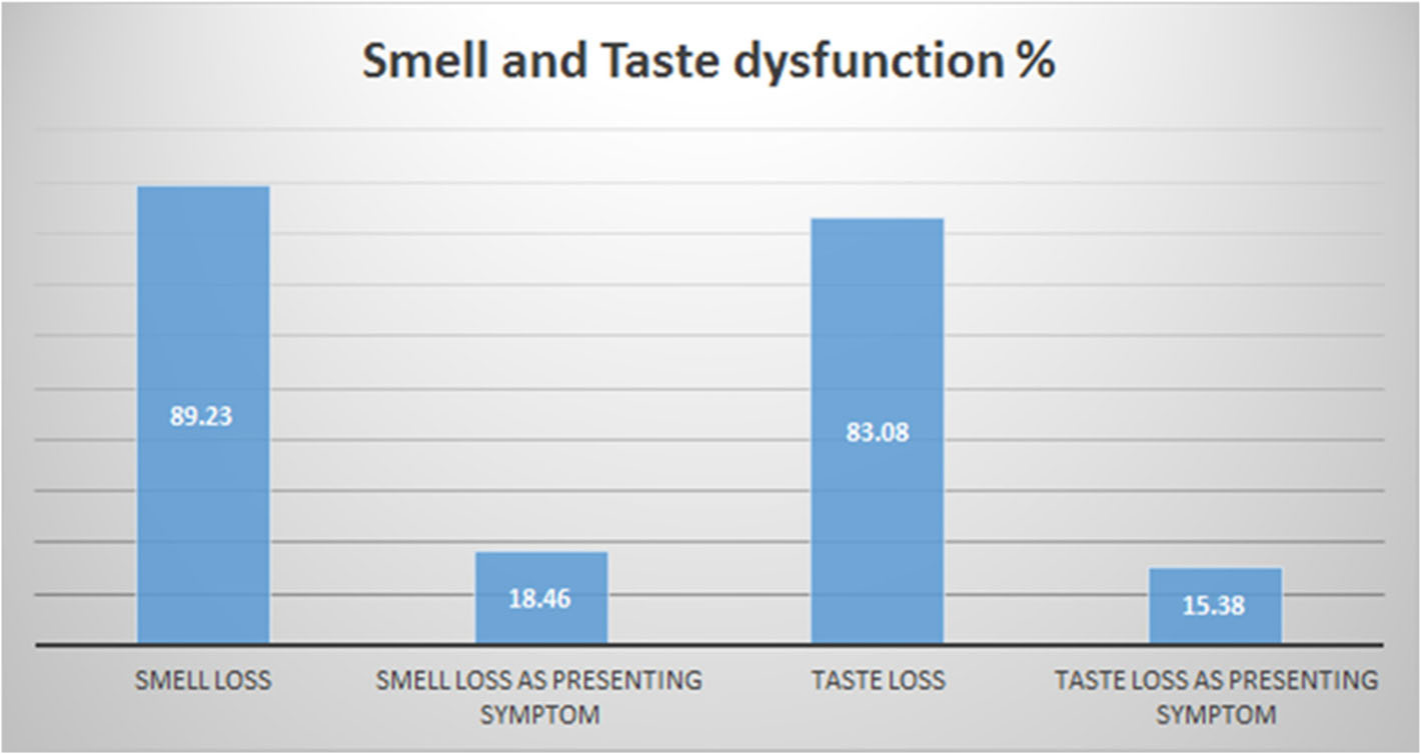
The percentage of smell and taste loss in general and as the only presenting symptom of COVID-19

Smell dysfunction appeared in 58/65 (89.23%) patients with or without other symptoms of COVID-19; twelve of them had been tested for COVID-19 because they were in contact with other positive symptomatic patients. All these 12/65 (18.46%) patients confirmed that they had no symptoms other than loss of sense of smell before they were tested for COVID-19, and 10/65 (15.38%) patients had taste loss in addition (Fig. 1).

Taste dysfunction was found in 54/65 (83.08%) patients with other COVID-19 symptoms. Only 16/54 (29.63%) of them recovered.

**Fig. 2:**
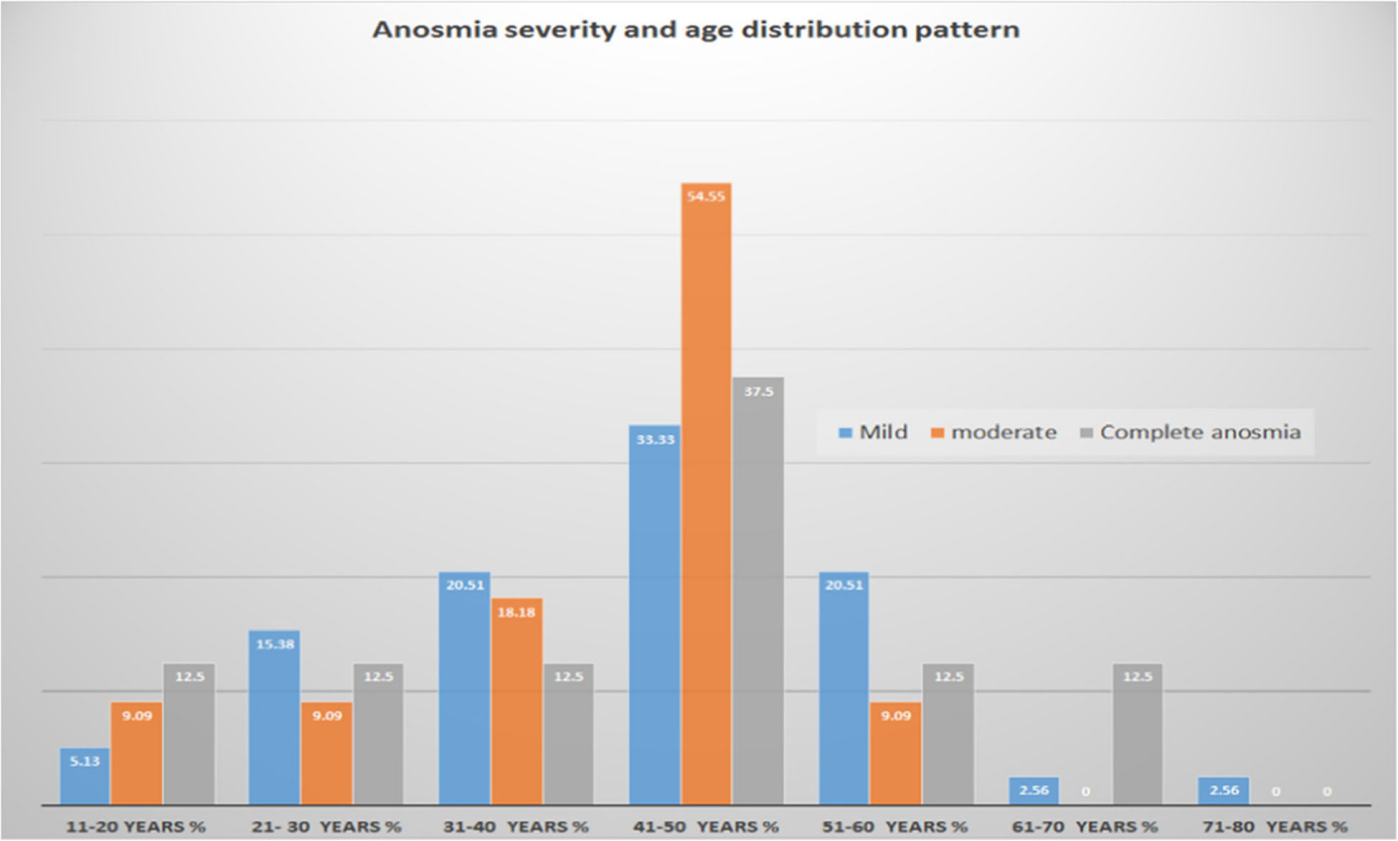
The percentage of smell loss severity in the different age groups

**Fig. 3:**
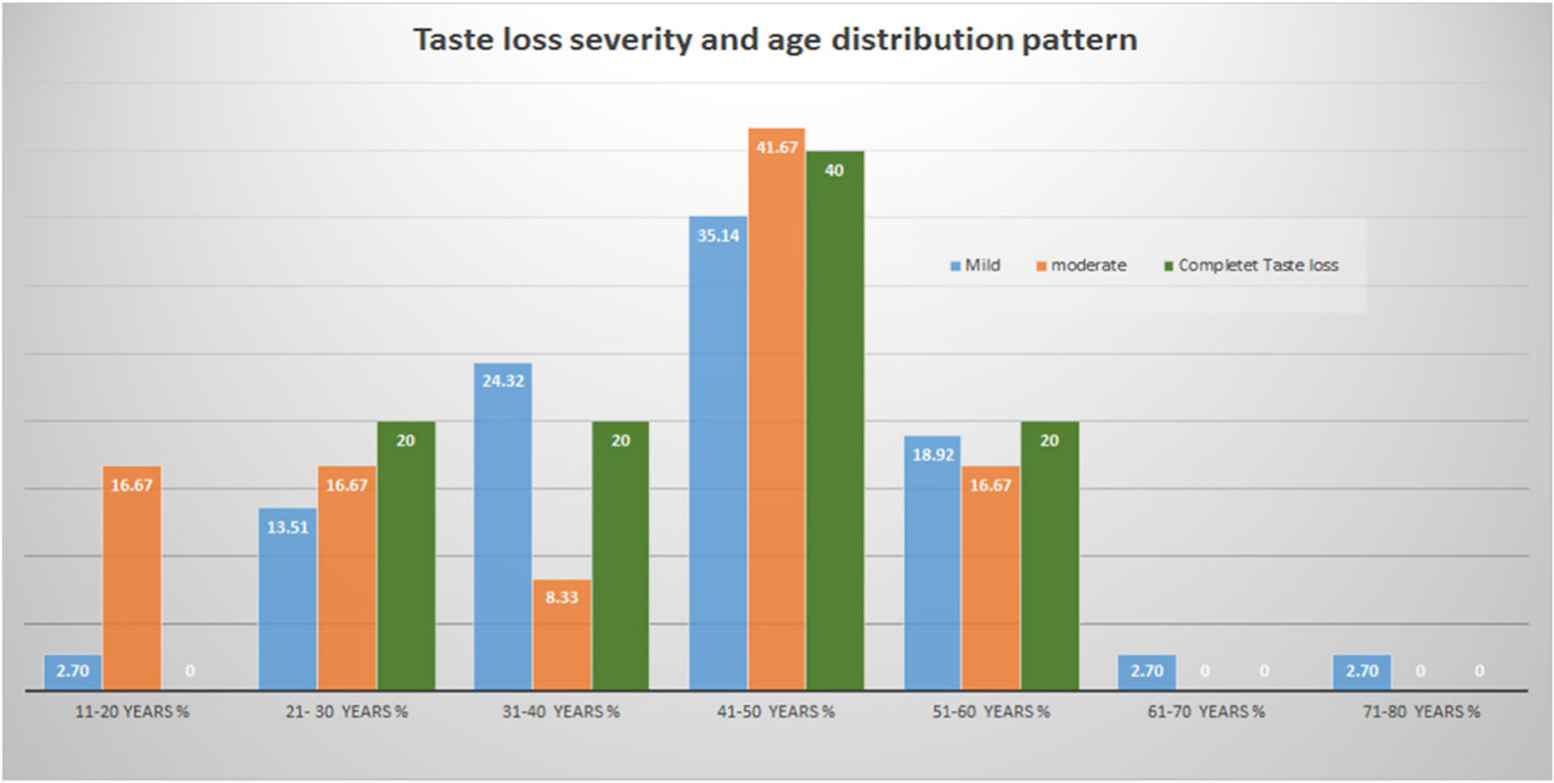
The percentage of taste loss severity in the different age groups

Anosmia was mild in 39/58 (67.24%) patients; of them, 16/39 (41.03%) were male, and 23/39 (58.97%) were female. Eleven of 58 (18.97%) had moderate anosmia; of them, 5/11 (45.45%) were male, and 6/11 (54.55%) were female. Eight of 39 (20.51%) had complete anosmia, 3/8 (37.5%) were males and 5/8 (62.5%) females (Tables 1 and 2; Figs. 2 and 3).

**Table 1:** Anosmia severity gender distribution

Anosmia severity	Number	Number %	Male	Male %	Female	Female %
Mild	39	67.24	16	41.03	23	58.97
moderate	11	18.97	5	45.45	6	54.55
Complete anosmia	8	13.79	3	37.5	5	62.5
Total no.	58	100

**Table 2:** Taste loss severity gender distribution

Taste loss severity	number	*n*%	Male	Male %	Female	Female %
Mild	37	68.52	15	40.54	22	59.46
Moderate	12	22.22	7	58.33	5	41.67
Complete taste loss	5	9.26	2	40.00	3	60.00
Total no.	54	100.00

The timing of smell loss appearance in relation to the COVID-19 confirmation by PCR ± CT scan was only 1/58 (1.72%) after the confirmation. However, appearance before confirmation had variable timing. Twelve of 58 (20.68%) appeared within less than a week before confirmation, while 18/58 (31.03%) appeared before 1 week. In addition, some patients took a longer time between smell loss and confirmation; 19/58 (32.76%) of them took 2 weeks, 6/58 (10.34%) took 3 weeks, and 2/58 (3.45%) 1 month or more.

Twenty-three of 58 (39.66%) patients had the sense of smell recovered. The recovery took 1–3 weeks to occur. The time taken to recover the smell distributed as 21.74% recovered within less than a week, 52.17% 1 week, 21.74% within 2 weeks, and 4.35% 3 weeks.

**Fig. 4:**
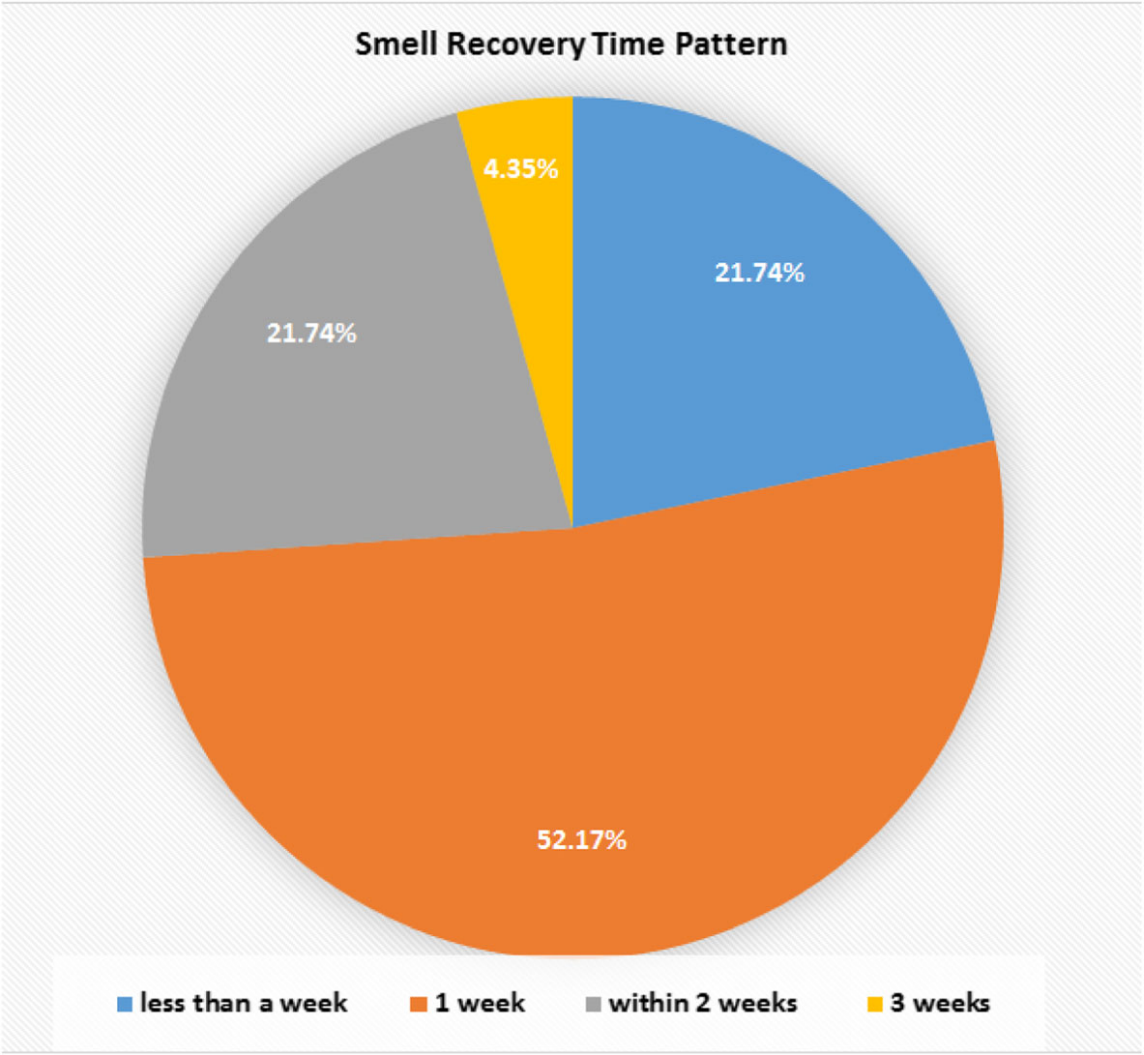
The percentage of smell recovery in the corresponding periods

The timing of taste loss appearance in relation to the COVID-19 confirmation was only 4/54 (7.41%) after the confirmation. Appearance before confirmation had variable timing also as in smell. Where 4/54 (7.41%) had taste loss within less than a week before confirmation, 18/54 (33.33%) before 1 week, 15/54 (27.78%) before 2 weeks, 10/54 (18.52%) before 3 weeks, and 3/54 (5.56%) 1 month or more before confirmation (Fig. 4).

**Fig. 5:**
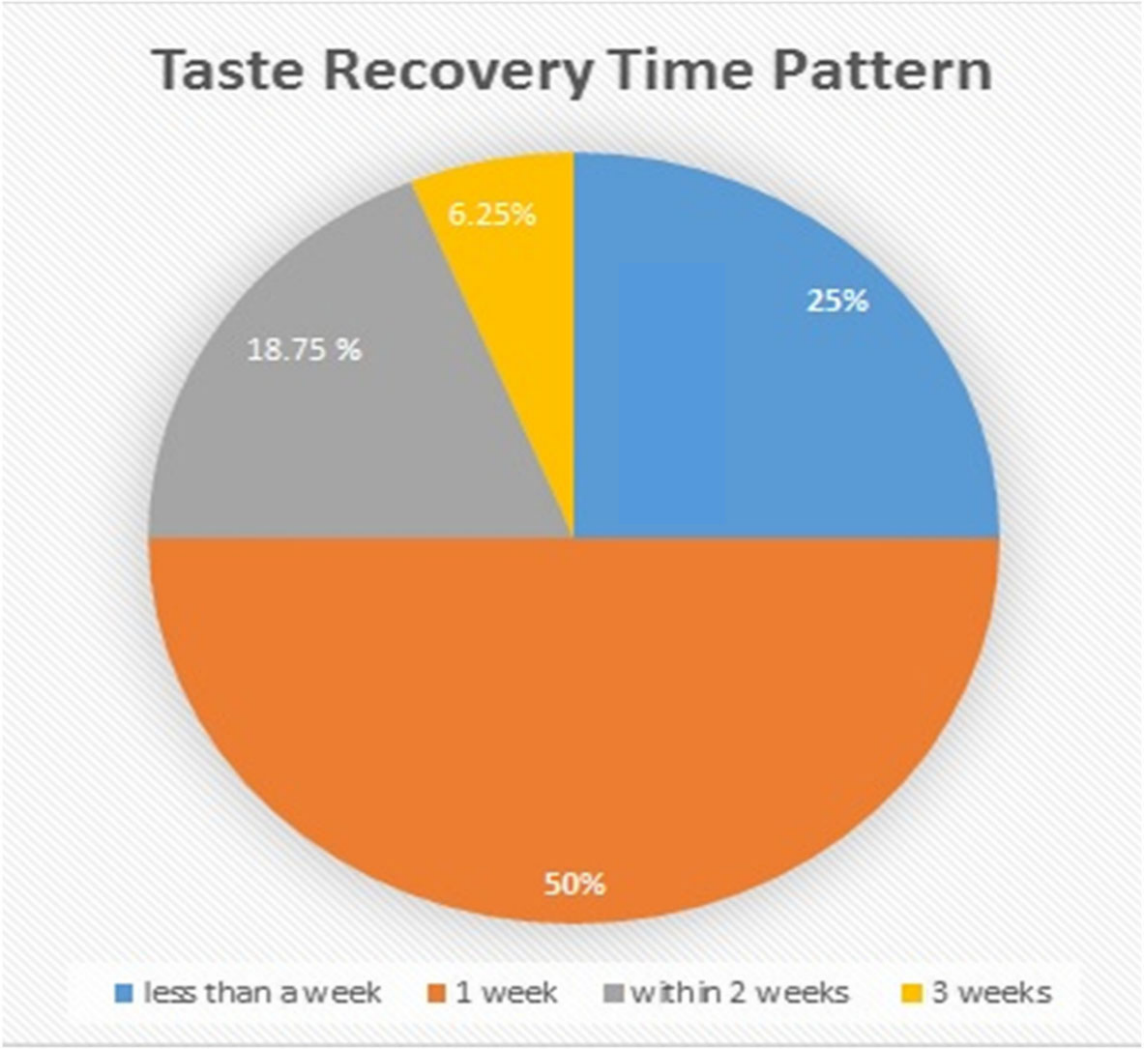
The percentage of taste recovery in the corresponding periods

The time for taste recovery distributed as 25% recovered within less than a week, 50% in a week, 18.75% within 2 weeks, and 6.25% in 3 weeks (Fig. 5).

**Fig. 6:**
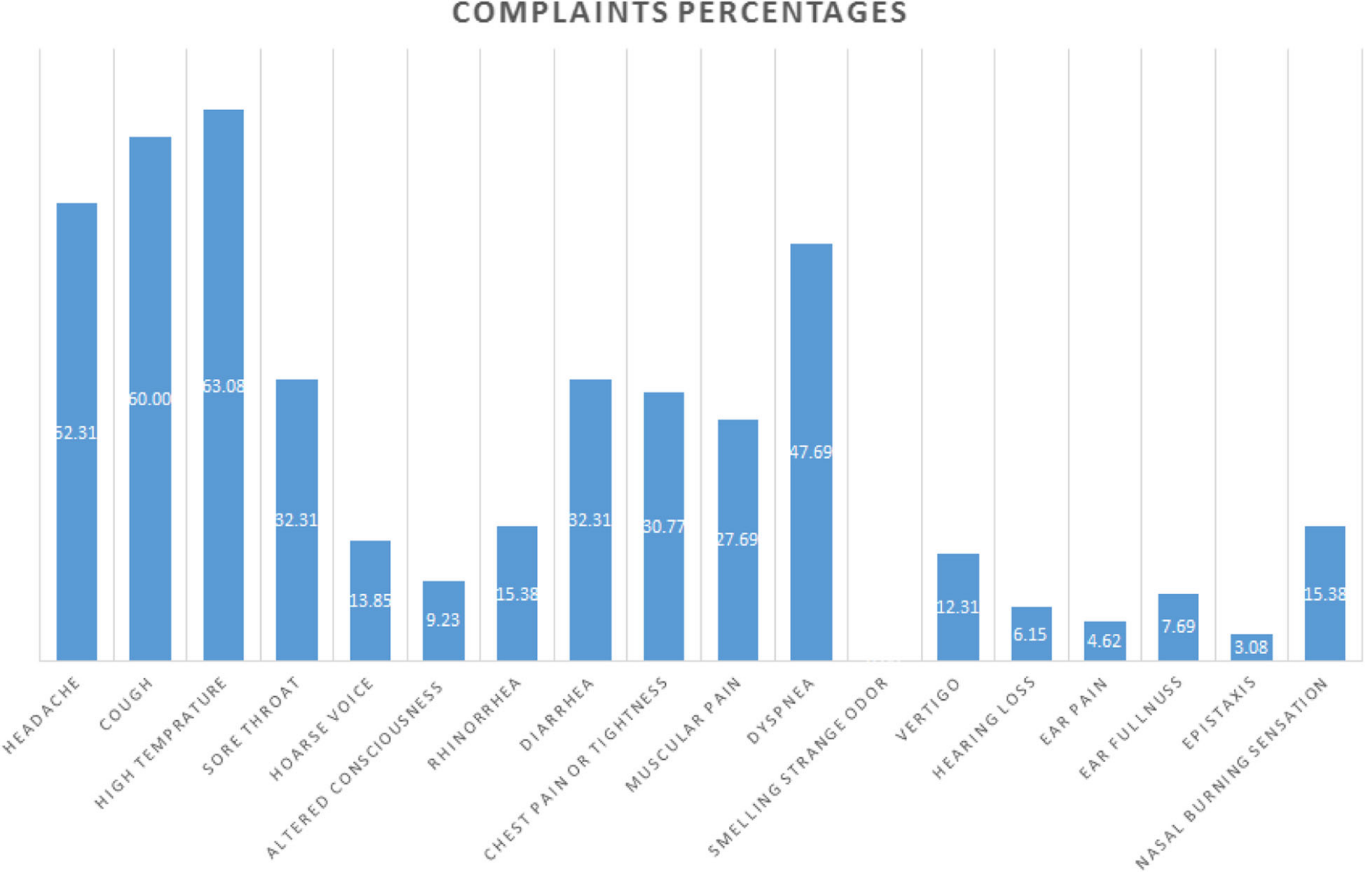
The percentage of each symptom in COVID-19 patients

The most common complaints were high temperature (63.08%), cough (60.00%), headache (52.31%), dyspnea (47.69%), both sore throat and diarrhea (32.31%), and chest pain or tightness (30.77%). Figure 6 show the percentage of different patients’ complaints.

Fifty-seven patients (87.69%) were non-smokers, while only eight (12.31%) patients were smokers.

## Discussion

Viral infections are common causes of loss of smell, and many cases of transient loss may be missed [5]. Over the last 2 months, an increase of sudden isolated anosmia incidence, with or without taste loss, was reported. In this study, chemosensitive dysfunction appeared in high percentage of the COVID-19 patients, 89.23% of patients had smell dysfunction and 83.08% had taste dysfunction [2, 6].

Sudden isolated chemosensitive dysfunction appeared in 18.46% and 15.38% for anosmia and taste loss, respectively. Interestingly, these percentages were reported in patients who were tested because of only contact with COVID-19 relative; two of them reported the loss of smell as the first symptom, while other 10 patients reported no first complaint except contact with COVID -19 patient, and when it comes for the symptoms questionnaire, they had reported only chemosensitive dysfunction.

Females gender was more affected in this study, although with little difference. This may be because many male patients refused giving consent to participate. However, other studies reported this gender percentage [5–8].

Most cases were mild, followed by moderate, then total anosmia and or taste loss. There must be recognition of the patient feeling to have chemosensitive disorders and the real presence of such dysfunction, i.e. the subjective and objective chemosensitive dysfunction. More subjective studies that investigate the taste and smell dysfunction are needed, although it is difficult to conduct such study because the risk of spread of infection.

The age group distribution shows most chemosensitive dysfunctions were in the fourth decade and around it [9].

Regarding timing of chemosensitive dysfunctions in relation to the confirmation of COVID-19, more than half of patients with dysfunction appeared 1 week or less before confirmation, which may give a clue to the cause of this dysfunction.

Recovery of chemosensitive dysfunction occurred within 1–3 weeks; most of them recovered within the first week [2]. This means the dysfunction is transient in most of the cases and reversible. Most of them did not take specific treatment for the chemosensitive dysfunction.

Majority of patients were non-smokers as shown by other studies [2, 10]. The relation of smoking to COVID-19 is interesting and needs to be investigated in further studies.

## Conclusion

Chemosensitive dysfunction is associated with coronavirus disease and may be the only symptom that presents the disease. This makes the ENT doctors in the first line of contact with coronavirus. Further objective studies with larger sample are required to cover chemosensitive dysfunctions, as the recognition of this dysfunction may help the COVID-19 diagnosis, and prevent the spread of this disease.

## Data Availability

The authors confirm that data and materials are available.

## Abbreviations

COVID-19: Coronavirus disease 2019
HCoV OC43: Human coronavirus (strain OC43)
OD: Olfactory dysfunction
URTI: Upper respiratory tract infection
